# A humble neuroanatomist: Martin Berry, PhD (1936–2021)

**DOI:** 10.1111/ejn.15681

**Published:** 2022-05-06

**Authors:** Zubair Ahmed, Ann Logan

**Affiliations:** ^1^ Institute of Inflammation and Ageing, College of Medical and Dental Sciences University of Birmingham Birmingham UK; ^2^ Division of Biomedical Sciences University of Warwick Coventry UK

Martin Berry (Figure 1), Emeritus Professor of Neuroanatomy at the University of Birmingham and a former Chairman of the Department of Anatomy, Kings College London (Guys Campus), died on 30 July 2021 after a valiant fight with cancer. He was 85 years old. The field of Neuroscience has lost a great and humble visionary.

**FIGURE 1:**
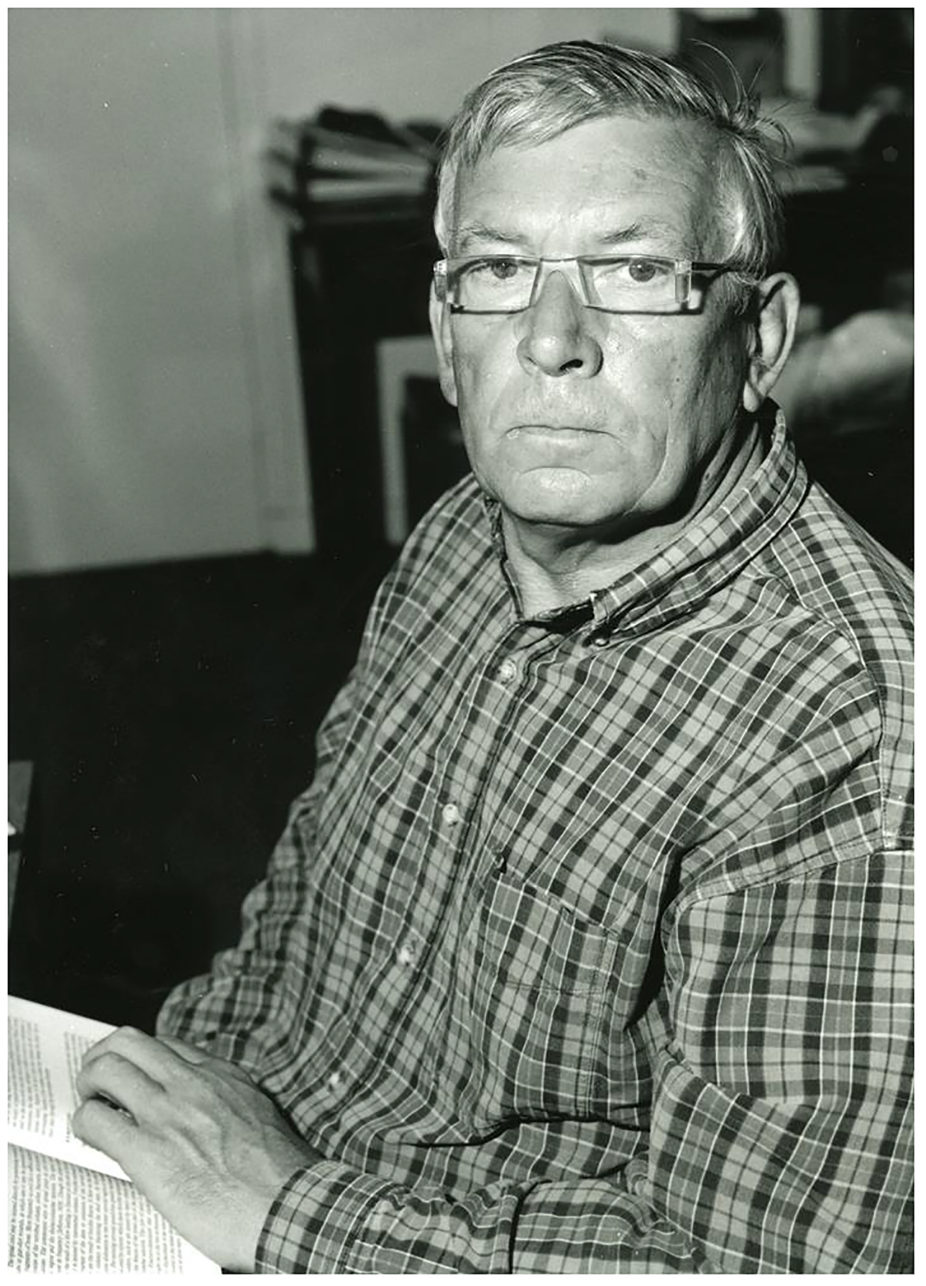
Photograph of Professor Martin Berry

Martin studied Medicine at the University of Birmingham, but whilst studying, he earned an intercalated BSc degree (1961) followed by a PhD in Neuroanatomy (1964) and eventually graduated with an MBChB in 1967. Having trained as a General Practitioner and spending a brief spell working as one, his love of science overtook him, and he returned to academia in 1969 taking up a post as Lecturer, then Senior Lecturer, then Reader, and in 1982, took up a post as Chair and Head of the Department of Anatomy at Kings College London (Guys Campus). He retired in 2001 but came back to the University of Birmingham as an Emeritus Professor of Neuroanatomy. He continued to teach and took an active part in research right up until when he passed away.

Martin was an innovator who was always ahead of the field. His bold and original ideas inspired many scientists across the globe. His early work focused on histogenesis of the cerebral cortex and he showed that the normal six layered cortex was established by inward migration of cells, ultimately constituting to layers V and VI, intermingled with an outward migration of cells destined to form layers II, III and IV (Berry et al., 1964; Berry & Eayrs, 1963). Later, Martin identified the major NG2‐expressing glia in the adult CNS as a distinct class of cells, calling them ‘synantocytes’ ('synanto' meaning ‘contact’), since they formed multiple contacts to neurons, astrocytes, oligodendrocytes and myelin (Berry et al., 2002).

However, Martin's seminal contributions in the field of CNS axon regeneration is what set him out as a true visionary and inspired many to follow in his stead. For example, it was known that the projection cells of the retina, the retinal ganglion cells (RGC) could regenerate their axons through the environment of a peripheral nerve graft affixed to the cut end of the optic nerve (David & Aguayo, 1981). However, axon regeneration through the native environment of the optic nerve was long considered to be impossible due to the hostile inhibitory nature of the optic nerve. Martin was the first to demonstrate that the RGCs could regenerate their axons through the native environment of the optic nerve after a crush injury, if a small piece of sciatic nerve was implanted into the back of the eye (Berry et al., 1996). This was a revolutionary discovery demonstrating that RGCs could regenerate their axons through the native inhibitory environment of the optic nerve, sparking a renewed enthusiasm in the field of CNS axon regeneration.

He continued his work in CNS axon regeneration, even after his retirement in 2001, being the driving force in some of the key observations in the field. These include demonstrating how scar tissue was laid down at a CNS injury site and the important role of TGFβ1 and β2 in CNS scar formation (Logan & Berry, 1993), how RGC axon regeneration promoted by peripheral nerve implants inserted into the back of the eye, stimulated astrocytes in the optic nerve to secrete matrix metalloproteases (MMPs) to facilitate axon regeneration (Ahmed et al., 2005) and how Schwann‐cell‐derived factors in a peripheral nerve implant promoted RGC axon regeneration through regulated intramembranous proteolysis (RIP) of p75^NTR^, the main axon growth inhibitory signalling molecule (Ahmed et al., 2006; Logan et al., 2006).

In 2011, Martin guided the studies that led to the discovery that caspase‐2, always thought of as an initiator caspase, could actually take the function of an executioner caspase and thus kill RGC (Ahmed et al., 2011). After optic nerve crush injury, RGC exclusively activated caspase‐2 and a short interfering RNA molecule, designed to resist nucleases degradation, promoted survival of over 98% of RGC within 7 days (Ahmed et al., 2011) with later studies showing neuroprotection of >95% of RGC after 21 days (Vigneswara et al., 2014).

Martin was a very charismatic, nonchalant and humble neuroanatomist. He was very well liked and took the time to get to know everyone around him. When he spoke, he did so with authority, knowledge and integrity. Martin was an extremely passionate scientist, an inspirational mentor with an endless inquisitive mind. His scientific curiosity was boundless and he had a remarkable grasp of the ‘nitty gritty’ of the different pathways involved in axon regeneration. His knowledge was so remarkable that after speaking to him you were left wondering how a person can fathom all of the innuendoes involved and make ‘head or tail’ of anything. His appetite for neuroscience was infectious, he was always full of big ideas, and his irreproachable style touched the lives of so many friends, students and collaborators.

Every person that knew Martin viewed him as a unique individual who was kind, honest, generous, extremely humble and a very thoughtful man. It is fitting, given the focus of his research on the visual system, that he illuminated the field and brought clarity through his discoveries. Martin supported many trainees and faculty members throughout their career and helped them to step up and take charge of their own areas of research by providing a nurturing and evidence‐based research environment. Martin lived for research, as demonstrated by his commitment even though he retired at the age of 65. He would still come into the lab every day, perform experiments, analyse data, assimilate information, write papers, teach, mentor and supervise. Despite being retired, he never lost the ‘hunger’ for the next discovery and was always eager to help. He was an exemplar of perseverance, persistence and perfection. His remarkable knowledge of neuroanatomy, together with his powers of recall and his ‘big personality’ will be greatly missed by all those who knew him. He has left a significant void, both scientifically and personally, that it will be impossible for anyone to fill. However, we will do our utmost to remember his huge contributions to our lives and appreciate and emulate his wonderful attributes.

Martin is survived by his wife, and their two daughters and two sons. He will be dearly missed by those in the College of Medical and Dental Sciences of the University of Birmingham and the entire Neuroscience community.

## CONFLICT OF INTEREST

The authors declare that they have no competing interests.

### PEER REVIEW

The peer review history for this article is available at https://publons.com/publon/10.1111/ejn.15681.
